# The evaluation of athletic footwear on postural stability in older adults: an exploratory study

**DOI:** 10.1186/1757-1146-4-S1-O11

**Published:** 2011-05-20

**Authors:** Angela Brenton-Rule, Annie Walsh, Sandra Bassett, Keith Rome

**Affiliations:** 1 School of Podiatry, Health & Rehabilitation Research Institute, AUT University, Auckland, New Zealand; 2 School of Physiotherapy, Health & Rehabilitation Research Institute, AUT University, Auckland, New Zealand

## Background

A decrease in postural stability may increase the risk of falling in the older adult. The relationship between athletic footwear and postural stability has been reported in previous studies with conflicting results. The aim of the current study is to evaluate differences between two different types of athletic footwear and barefeet, in relation to postural stability in asymptomatic older adults.

## Methods

Twenty-one older adults (mean: 74 SD: 5 years) were recruited from a University-based clinic. The cross-sectional study evaluated two different walking shoes (shoe 1: ASICS Gel Odyssey^™^; shoe 2: ASICS Cardio Velcro^™^) and barefoot. Participants gave informed consent and attended a laboratory setting where they carried out standard tests of quiet standing balance of 30 seconds duration on a Tekscan Matscan® pressure mat. Each participant performed three repetitions of bipedal standing with eyes open and eyes closed under three randomised conditions. Two-way, repeated measures, within-groups Analysis of Variance (ANOVA) examined significant differences between the three footwear conditions and two vision conditions in terms of postural sway in quiet standing. Postural sway was measured as centre of pressure excursions in an anterior-posterior (AP) and medio-lateral (ML) direction (cm).

## Results

The results demonstrated a significant difference (p<0.05) in AP postural direction with eyes open between barefoot (Mean: 1.39; SD: 0.58cm) and shoe 1 (Mean: 1.76; SD: 0.48cm); and shoe 2 (Mean: 1.74; SD: 0.52cm). The results also demonstrated a significant difference in AP postural direction (p<0.05) with eyes closed between barefoot (Mean: 1.72; SD: 0.58cm) and shoe 1 (Mean: 2.25; SD: 0.86cm); and shoe 2 (Mean: 2.31; SD: 0.81cm). No significant difference in mean ML postural direction between the two footwear conditions and barefoot, with eyes open and eyes closed was found (p>0.05).

## Conclusions

In both of the walking shoes, when standing with eyes open and eyes closed, AP sway range was significantly increased when compared to barefeet. The results suggest that older adults demonstrate an initial destabilisation effect, which could possibly be of benefit to functional ability over a longer duration. The potential of athletic footwear to enhance postural stability requires further long-term investigation.

